# Subcutaneous Pectoral Edema After Arthroscopic Labral Repair Despite Normal Irrigation Fluid Usage and Surgery Duration

**DOI:** 10.1111/os.14324

**Published:** 2025-01-15

**Authors:** İnci Hazal Ayas, Yağız Oğul Akcan, Miray Haspolat, Mehmet Ali Tokgöz, İlke Keser, Ulunay Kanatlı

**Affiliations:** ^1^ Department of Physiotherapy and Rehabilitation Faculty of Health Sciences, Gazi University Ankara Turkey; ^2^ Department of Orthopaedics and Traumatology Faculty of Medicine, Gazi University Ankara Turkey

**Keywords:** postoperative complications, shoulder arthroscopy, subcutaneous edema

## Abstract

**Objectives:**

Edema after shoulder arthroscopic surgery poses concerns due to its potential complications such as compartment syndrome, nerve damage, and respiratory issues. This study aimed to investigate the acute accumulation of subcutaneous fluid after shoulder arthroscopy.

**Methods:**

A prospective cohort study, providing Level III evidence was conducted on 50 patients undergoing arthroscopic shoulder surgery under interscalene block anaesthesia from September to December 2023. The patients were divided into two groups: rotator cuff repair (RCR, *n* = 29) and labral repair for shoulder instability (LR, *n* = 21). Subcutaneous fluid levels were measured preoperatively, postoperatively, and at discharge (24 h postsurgery) using the MoistureMeterD Compact at the neck, pectoral region, deltoid area, cubital fossa, and carpal tunnel. Data on surgery duration and irrigation fluid volume were documented. In the statistical analysis, repeated measures ANOVA and the independent‐samples *t*‐test were applied to compare parametric data, while the Friedman test and Mann–Whitney *U* test were used for nonparametric data.

**Results:**

The average operation time was 29.0 ± 12.1 min for RCR and 30.0 ± 10.9 min for LR, with average irrigation fluid use of 3.8 ± 0.9 and 4.0 ± 0.7 L, respectively (both *p* > 0.05). There was no increase in subcutaneous edema in the neck and deltoid region in both groups. At discharge, the percentage of subcutaneous fluid increased in the cubital fossa (*p* = 0.04 for RCR; *p* < 0.001 for LR) and carpal tunnel (both *p* < 0.001) in oth groups, whereas pectoral edema increased only in the labral repair group (*p* = 0.04).

**Conclusions:**

Subcutaneous pectoral edema can occur following arthroscopic labral repair, and increased fluid levels in the cubital fossa and carpal tunnel were observed in both rotator cuff and labral repairs, even in the absence of prolonged surgery or excessive irrigation fluid use. These findings highlight the need for careful postoperative management of edema after shoulder arthroscopy, particularly for labral repairs, with special attention to the pectoral region, cubital fossa, and carpal tunnel to prevent potential complications.

The registry is sponsored by the United States National Library of Medicine (www.clinicaltrials.gov); Registry Name: Examination of Edema After Arthroscopic Shoulder Surgery ID: NCT06014203.

## Graphical abstract

Subcutaneous pectoral edema can occur following arthroscopic labral repair, with increased fluid levels also observed in the cubital fossa and carpal tunnel after both rotator cuff and labral repairs. Despite normal irrigation fluid usage and surgery duration, these findings highlight the importance of careful postoperative edema management to prevent potential complications.

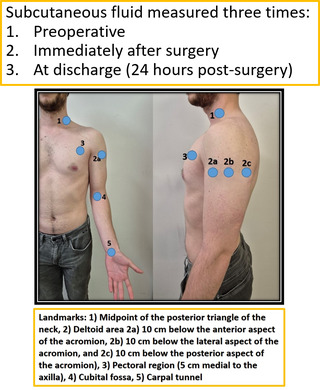

## 1 Introduction

Orthopedic surgeries are often associated with extensive damage to soft tissues and lymphatic vessels. Severe fractures and knee and hip joint replacements can disrupt lymphatics, leading to chronic edema, muscle weakness, and slower recovery [1]. Similarly, upper extremity surgeries can also cause prolonged swelling, affecting joint range of motion, tissue mobility, scar formation, function, strength, and appearance. These complications can delay recovery, return to work, daily activities, and increase the need for outpatient appointments [1]. In addition to orthopedic surgery, being a trauma itself and a risk for edema formation, fluid extravasation also occurs after arthroscopic surgeries.

Shoulder arthroscopic surgery typically refers to the leakage of fluid used during the surgical procedure into the surrounding tissues, and while edema is a common postoperative occurrence, fluid extravasation presents a more serious risk with potential for severe complications [2, 3]. During arthroscopic surgery, the surgeon usually employs a saline solution to irrigate the joint, which aids in improving visualization and removing debris. Fluid extravasation following shoulder arthroscopic surgery is a concern due to its potential implications for postoperative complications and patient outcomes. While a certain amount of fluid is expected to resolve spontaneously within 24 h, excessive extravasation can lead to complications such as compartment syndrome, nerve damage, and respiratory or cardiovascular issues [2, 3]. Peripheral neuropathy, particularly common after shoulder surgery, is one such complication [4, 5]. The increase in subcutaneous fluid in the distal extremity postsurgery may contribute to the development of compressive peripheral neuropathy. Chest wall swelling and neck swelling are also reported as the most common symptoms of fluid extravasation after shoulder arthroscopic surgery [2]. Although it is known that these symptoms reported after shoulder arthroscopic surgery are associated with excessive fluid use and prolonged surgery time, there is no reported information regarding the type of surgery, such as labral, or rotator cuff surgery [2, 3]. Examining subcutaneous fluid accumulation based on the type of surgery may enhance our understanding of postoperative complications such as edema, carpal tunnel syndrome, compartment syndrome, myolysis, and dyspnea. Although no prior studies have specifically employed quantitative analysis for subcutaneous edema in the axilla, peri‐axillary regions, and arm following shoulder arthroscopy, there are case reports that have evaluated chest and neck edema through observation and circumference measurements [6, 7]. Our study utilizes tissue dielectric constant (TDC) measurements with the MoistureMeterD device, an approach that has been previously used for assessing localized tissue water in patients with and without lymphedema in the arm and neck [8, 9]. Subcutaneous edema often signifies fluid accumulation that extends from deeper tissues, indicating a broader physiological response to injury, inflammation, or surgical intervention. Edema in the axillary and peri‐axillary regions, as well as the arm, following shoulder arthroscopy can compress surrounding structures and impede venous return.

The aims of this study are: (i) to investigate the acute accumulation of subcutaneous fluid following shoulder arthroscopic surgery, (ii) to compare the fluid distribution patterns between patients undergoing rotator cuff repair (RCR) and those undergoing labral repair (LR) for shoulder instability, and (iii) to determine whether certain types of shoulder arthroscopy are associated with higher risks of fluid extravasation and related complications, in order to guide postoperative monitoring and management strategies. The study hypothesizes that subcutaneous fluid accumulation and edema patterns will differ between patients undergoing RCR and those undergoing LR.

## 2 Materials and Methods

### 2.1 Study Design and Participants

The study, planned as a prospective cohort study, was registered on ClinicalTrials.gov with the prospective study registration number NCT06014203. It received ethical approval from the Gazi University Ethics Commission on September 4, 2023, with the application number 042d5901, and obtained informed consent from all participants. This study investigated the subcutaneous fluid accumulation during RCR and LR for shoulder instability procedures at various anatomical points before, immediately after, and 24 h following shoulder arthroscopic surgery. The study included patients who underwent arthroscopic shoulder surgery between September 2023 and December 2023 in the Orthopedics and Traumatology Clinic of the Faculty of Medicine Hospital affiliated with the authors. Fifty patients who agreed to participate in the study and met the inclusion criteria were included.

### 2.2 Inclusion and Exclusion Criteria

The inclusion criteria for patients were: (1) Patients aged between 18 and 65 years, (2) Patients scheduled for arthroscopic RCR or LR for shoulder instability. Exclusion criteria for patients were: (1) Patients with any metabolic and endocrine diseases, (2) Patients with previous shoulder surgery (3) Patients with stiff shoulder, (4) Patients with any dermatological pathologies, and (5) Patients with hyperlaxity.

### 2.3 Surgical Procedure

All patients were operated on by a single surgeon under interscalene block anesthesia, using the lateral decubitus position with the arm at 45° of abduction and 15° of flexion under 4 kg of longitudinal traction on a shoulder holder. A standard bandage dressing was used around the forearm and upper arm to ensure patient safety and maintain limb stability during surgery. During arthroscopy, an arthro‐pump (FMS VUE II System, DePuy Mitec Inc.) with 50 mmHg pressure was employed. The volume of physiological saline fluid administered during the procedures was documented by the surgical team. All incisions were closed using standard sterile dressings without the application of additional pressure, to avoid compression that could potentially influence the postoperative assessment of edema. Furthermore, the type of surgery, the duration of each procedure, and the volume of physiological saline fluid administered were recorded by the surgical team performing the operations.

### 2.4 Measurement of Subcutaneous Fluid

**FIGURE 1:**
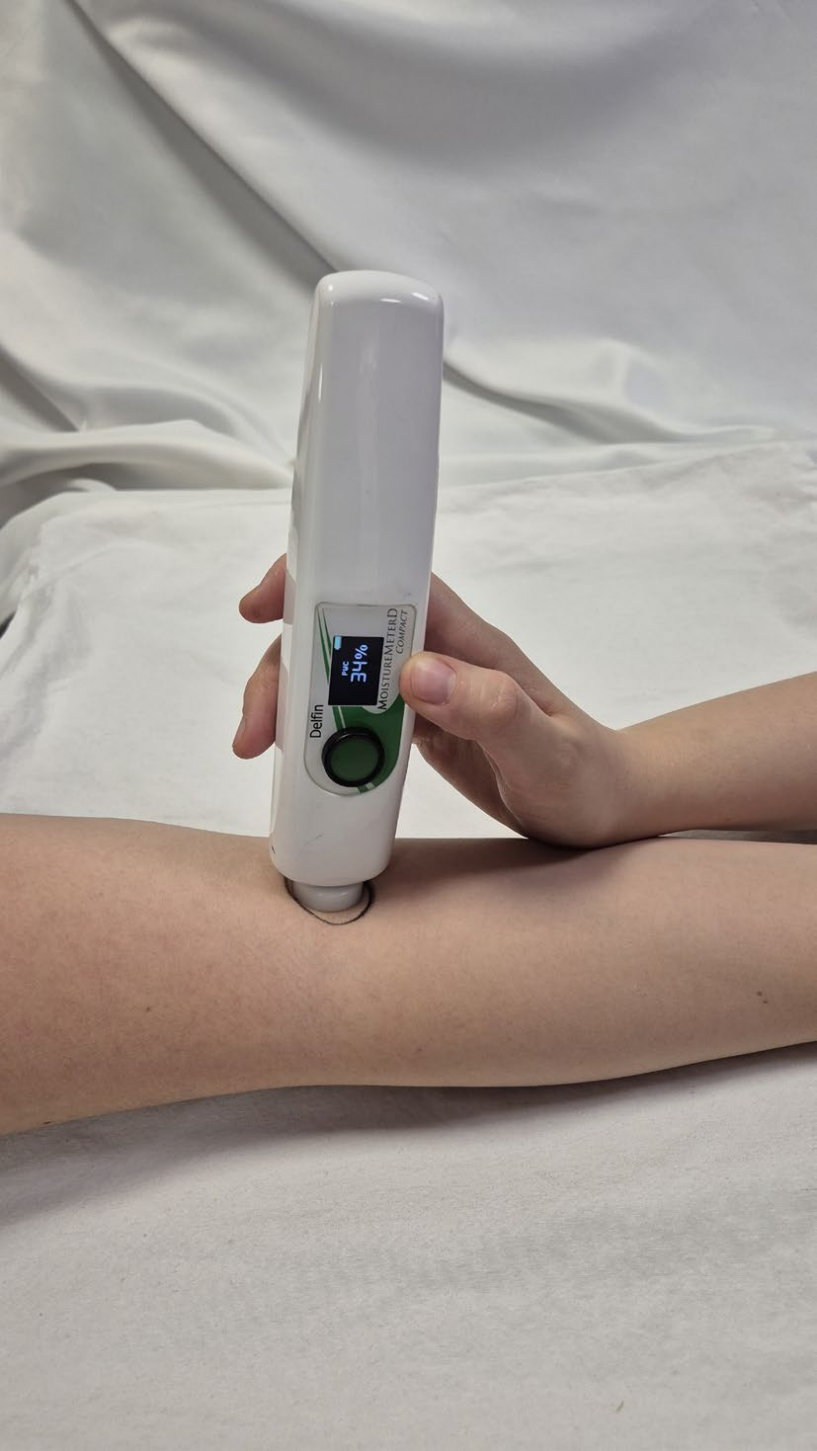
Measurement of cubital fossa edema using the MoistureMeterD Compact device.

**FIGURE 2:**
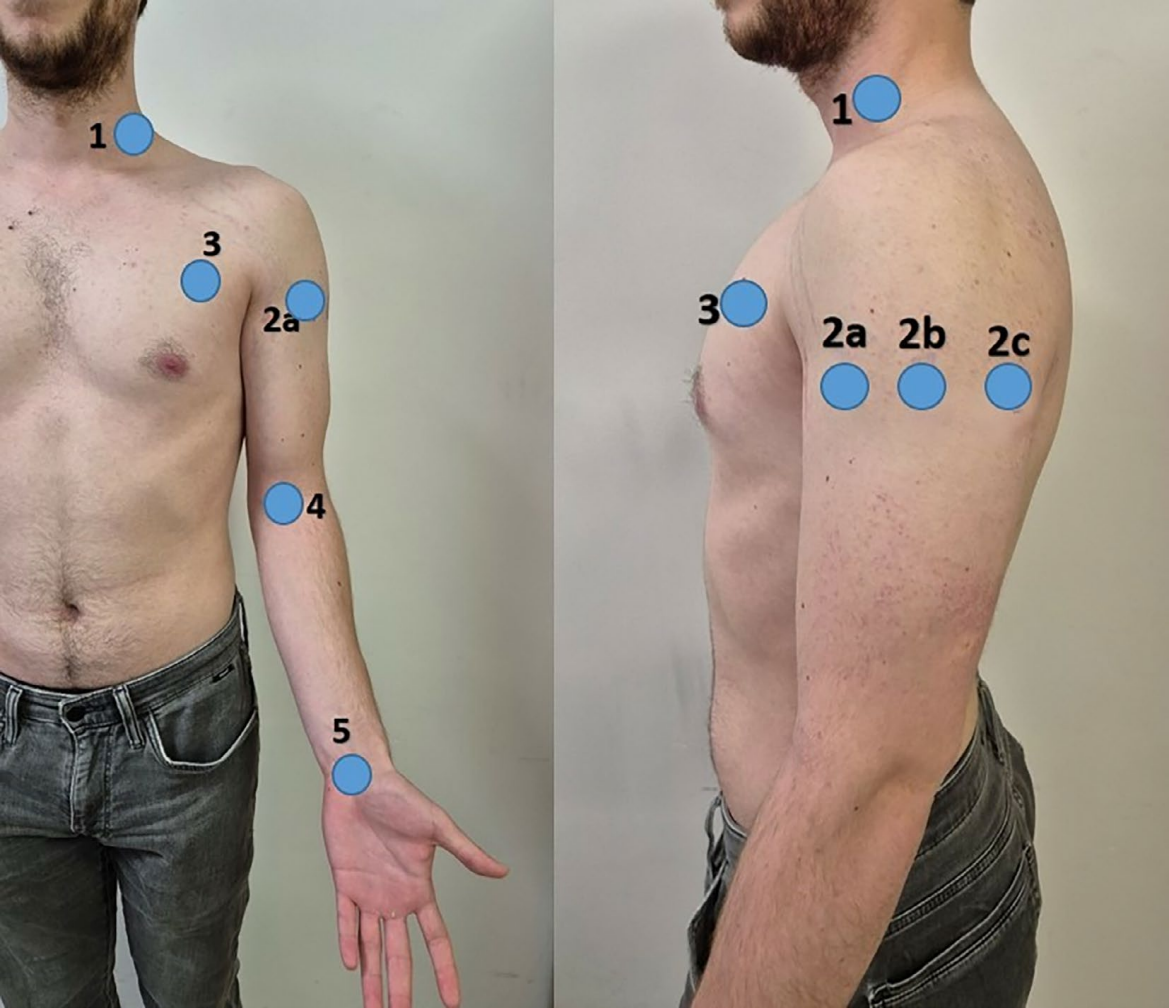
(1) Midpoint of the posterior triangle of the neck, (2) Deltoid area, (2a) 10 cm below the anterior aspect of the acromion; (2b) 10 cm below the lateral aspect of the acromion; and (2c) 10 cm below the posterior aspect of the acromion), (3) Pectoral region (5 cm medial to the axilla), (4) Cubital fossa, and (5) Carpal tunnel.

The extent of subcutaneous fluid in patients was measured three times in total: before surgery (preoperatively), immediately after surgery (postoperatively), and at discharge (24 h postsurgery) by a single researcher employing the MoistureMeterD Compact (Delfin Technologies, Finland, Figure 1), a local edema measurement device. The device measures the water content in biological tissues using the TDC on a percentage scale ranging from 0% to 100%, specifically emphasizing the subcutaneous layers at a depth of 2.5–5 mm. MoistureMeterD is a device used to measure tissue water in both lymphedema and nonlymphedema patients [9, 10] and it has intra‐ and inter‐reliability in local tissue water measurement in patients with lymphedema [10]. Several landmarks, determined by the researchers, were used for edema measurement: (1) Midpoint of the posterior triangle of the neck, (2) Deltoid area (divided into three parts: (a) 10 cm below the anterior aspect of the acromion, (b) 10 cm below the lateral aspect of the acromion, and (c) 10 cm below the posterior aspect of the acromion), (3) Pectoral region (5 cm medial to the axilla), (4) Cubital fossa, and (5) Carpal tunnel (see Figure 2). Before the surgery, each landmark was marked with a surgical pen by the researcher, who was also one of the authors, using a tape measure to ensure precise and consistent placement of the markings. Postoperative measurements were then taken at the same points. Three measurements were taken from each of the landmarks, and the average of these three measurements was used for statistical analysis. The difference in edema percentage, referred to as Δ edema, was calculated by subtracting the preoperative measurement value from the discharge measurement value (Δ = Discharge measurement—Preoperative measurement).

### 2.5 Postoperative Care and Rehabilitation

After surgery, all patients received standard postoperative care until discharge. This included immobilization with a neutral shoulder sling, cold pack application to the shoulder joint for 10 min every 3–4 h, and analgesics (paracetamol) for every 6 h. Upon discharge, patients were provided with home exercise‐based postoperative rehabilitation programs.

### 2.6 Statistical Analysis

According to the sample size analysis performed using the G*Power 3.1 analysis program, it was planned to include 50 patients in the study with 80% power and 5% type‐1 error [2]. All statistical analyses were performed using IBM SPSS Statistics Version 23 software (IBM SPSS Inc., Chicago, USA), and the data were expressed as mean ± standard deviation. The normality of the data was assessed using both visual methods (histograms and *Q*–*Q* plots), as well as analytical methods (Kolmogorov–Smirnov test). Parametric methods were used for measurement values suitable for normal distribution. In accordance with parametric methods, repeated measures one‐way ANOVA test (*F*‐table value) method was used to compare the measurement values. Nonparametric methods were used for measurement values that did not comply with normal distribution. In accordance with nonparametric methods, the “Friedman” test (*χ* ^2^‐table value) method was used to compare the measurement values. Comparisons between the RCR and LR groups were conducted using the independent‐samples *t*‐test for parametric data and the Mann–Whitney *U* test for nonparametric data. In statistical analysis, a *p*‐value lower than 0.05 was accepted as a significant difference.

## 3 Results

### 3.1 Patient Demographics and Surgical Data

The study included 29 patients undergoing RCR (59.3 ± 8.0 years) and 21 patients undergoing LR for shoulder instability (25.7 ± 5.2 years). The average surgery duration was 29.0 ± 12.1 min for RCR and 30.0 ± 10.9 min for LR, with irrigation fluid use averaging 3.8 ± 0.9 and 4.0 ± 0.7 L, respectively. The patients in the LR group were significantly younger (*p* < 0.001), but there was no difference between the groups in terms of gender, body mass index, operation time, or volume of irrigation fluid used (*p* > 0.05) (Table 1). None of the patients participating in the study experienced complications such as respiratory distress or distal neuropathy at the time of discharge.

**TABLE 1:** Patient's characteristics.

	Cuff repair, *n* = 29	Labral repair, *n* = 21	*p*
Gender (female)	55 (16)	47 (10)	0.15
Age (years) Min—Max	59.3 ± 8.0 43–73	25.7 ± 5.2 19–33	< 0.001*
BMI (kg/m^2^) Min—Max	28.9 ± 4.2 24.4–35.4	26,8 ± 5,8 19.7–35.0	0.33
Operation time (min) Min—Max	29.0 ± 12.11 14.0–56.0	30.0 ± 10.9 17.0–48.0	0.58
Fluid volume (mL) Min—Max	3862.0 ± 990.0 2250–5500	4045.8 ± 784.9 2250–5500	0.54

*Note*: Data presented as % (*n*) or mean ± SD. *Statistical significant (in bold).

Abbreviation: BMI, body mass index.

### 3.2 Subcutaneous Edema in the Pectoral Region

In the pectoral region, there was no increase in subcutaneous edema in the RCR group (*p* = 0.09), but there was an increase in the LR group (*p* = 0.04) (Table 2).

**TABLE 2:** Statistical analysis of preoperative, postoperative, and discharge measurements.

Location surgery		Preop measurement	Postop measurement	Discharge measurement	*F*/*χ* ^2^	*p*
Neck	Cuff repair	47.7 ± 5.1	47.9 ± 6.5	48.7 ± 5.1	2.1	0.13
	Labral repair	47.5 ± 4.9	49.0 ± 4.2	48.7 ± 3.6	1.0	0.34
Anterior acromion	Cuff repair	42.2 ± 7.4	42.3 ± 6.6	43.8 ± 5.5	1.5	0.22
	Labral repair	42.6 ± 7.4	42.0 ± 5.4	44.0 ± 6.3	0.9	0.37
Lateral acromion	Cuff repair	42.2 ± 5.4	44.6 ± 5.1	44.0 ± 5.0	0.3	0.67
	Labral repair	46.5 ± 4.2	45.0 ± 4.4	47.0 ± 5.3	2.3	0.11
Posterior acromion	Cuff repair	45.6 ± 5.1	45.3 ± 6.5	45.8 ± 6.2	0.1	0.79
	Labral repair	46.8 ± 5.7	47.1 ± 5.4	47.26 ± 4.4	0.1	0.89
Pectoral region	Cuff repair	44.3 ± 5.1	44.1 ± 5.0	45.2 ± 4.8	2.9	0.09
	Labral repair	43.1 ± 6.2	44.2 ± 5.5	46.2 ± 5.3	3.5	0.04*
Cubital fossa	Cuff repair	41.6 ± 6.1	43.1 ± 6.7	44.9 ± 7.4	3.4	0.04*
	Labral repair	38.5 ± 6.1	40.1 ± 6.9	45.1 ± 7.2	11.5	< 0.001*
Carpal tunnel	Cuff repair	45.7 ± 5.9	47.3 ± 6.5	53.4 ± 7.1	27.3	< 0.001*
	Labral repair	45.2 ± 6.9	48.4 ± 7.2	52.3 ± 6.5	12.4	< 0.001*

*Note*: Data presented as mean ± SD. *Statistical significant (in bold).

### 3.3 Subcutaneous Edema in the Cubital Fossa and Carpal Tunnel

In both groups, the percentage of subcutaneous fluid in the cubital fossa (*p* = 0.04 for RCR; *p* < 0.001 for LR) and carpal tunnel (both *p* < 0.001) increased significantly (Table 2).

### 3.4 Comparison of Edema Differences Between RCR and LR Groups

When comparing the difference in edema percentage (Δ edema = Discharge measurement—Preoperative measurement) between the RCR and LR groups, a significant difference was observed only in the pectoral region. The increase in edema in the pectoral region was significantly higher in the LR group than in the RCR group (*p* = 0.04) (Table 3).

**TABLE 3:** Comparison of the difference in edema percentage between rotator cuff repair and labral repair groups.

	Cuff repair	Labral repair	*p*
	Δ Edema (discharge—preop)	Δ Edema (discharge—preop)	
Neck	0.9 ± 5.2	1.1 ± 4.8	0.70
Anterior acromion	1.5 ± 5.9	1.4 ± 8.8	0.99
Lateral acromion	0.2 ± 5.6	0.4 ± 4.6	0.48
Posterior acromion	0.1 ± 4.3	0.4 ± 4.3	0.99
Pectoral region	0.9 ± 4.7	3.1 ± 5.7	0.04*
Cubital fossa	3.3 ± 8.0	6.5 ± 7.1	0.26
Carpal tunnel	8.6 ± 7.5	7.0 ± 9.9	0.59

*Note*: Data presented as mean ± SD. *Statistical significant (in bold).

## 4 Discussion

This study demonstrated that subcutaneous fluid accumulation in the cubital fossa and carpal tunnel occurs after shoulder arthroscopy for both RCR and LR. However, only the LR group exhibited significant pectoral region edema. Despite irrigation fluid volumes being well below the established safety threshold, fluid leakage was still observed. These results highlight the importance of monitoring subcutaneous fluid accumulation in patients undergoing shoulder arthroscopy, especially those receiving LRs, to reduce potential complications and enhance postoperative outcomes.

### 4.1 Fluid Accumulation and Its Implications

Several factors may contribute to fluid leakage during shoulder arthroscopy, including high pump pressure, large volumes of irrigation fluid, long operative times, the lateral decubitus position, obesity, advanced age, loose subcutaneous tissue, subacromial surgery, capsule resection, limited surgeon experience, and soft tissue or lymphatic vessel damage [1, 2]. The significant pectoral edema observed exclusively in the younger LR group suggests that fluid leakage is primarily influenced by procedural factors rather than patient demographics or the standardized pump pressure of 50 mmHg. Although there is no defined upper limit for irrigation fluid use, volumes over 20 L have been reported in symptomatic patients, with Memon et al. [2] deeming volumes below 20 L safe and acceptable. In our study, edema in the cubital fossa and carpal tunnel occurred even with fluid volumes within the safe limit. Although it is difficult to clearly explain the cause of this increase, we hypothesize that it may be due to the distal spread of irrigation fluid from the shoulder joint and/or the use of an immobilization sling after surgery. While it is recognized that traction, elevation, and bandaging applied during surgery may influence edema formation, elevation, and bandaging are typically applied to reduce edema. However, since the postoperative sterile dressing used in this study was pressureless, it is unlikely to have affected the accumulation of edema in the cubital fossa and carpal tunnel.

### 4.2 Surgical Technique and Fluid Distribution Patterns

Although there were no differences in operation time or irrigation fluid volume between the groups, only the LR group showed increased subcutaneous edema in the pectoral region. This could be due to the specific surgical techniques involved in LR compared with RCR. In LR for instability, the labrum is mobilized by lifting it from the glenoid using radiofrequency and a rasp under arthropump fluid pressure until the subscapularis muscle is visualized. In contrast, there is no labrum mobilization in RCR. We believe that this fundamental difference between the two surgeries accounts for the fluid leakage into the pectoral region observed in LR. Additionally, fluid accumulation in the peri‐axillary region during LR can lead to significant complications. Fluid in this area may exert pressure on surrounding structures, including nerves and blood vessels, which increases the risk of nerve damage and other issues. Since the axillary region houses critical neurovascular structures, increased fluid pressure can impair their function, potentially leading to pain, swelling, and even functional impairment of the upper limb. Interestingly, despite the irrigation fluid volumes in this study being well below the established safe limit, edema was still observed. Specifically, the average irrigation fluid use was approximately 4 L—about one quarter of the 20‐L threshold commonly cited as safe. This suggests that factors beyond fluid volume, such as the type of surgery and anatomical areas involved, may contribute to fluid accumulation. Therefore, monitoring fluid extravasation closely and considering additional preventive measures is crucial to mitigate complications like compressive neuropathies and other fluid‐related issues. This highlights the need for personalized postoperative care strategies that account for variability in patient responses to surgery.

### 4.3 Fluid Extravasation and Related Complications

A case report of shoulder arthroscopy for rotator cuff tear using interscalene block anesthesia and the lateral decubitus position described respiratory distress and stridor lasting 130 min after the use of 35 L of saline [11]. Similarly, Ercin et al. [6] reported pectoral swelling lasting 120 min after arthroscopic Bankart repair with 20 L of irrigation fluid. However, in our study, while pectoral edema occurred in patients who underwent LR, no respiratory complications were observed, likely due to the shorter surgery duration (maximum 56 min) and lower fluid use (maximum 5.5 L). Previous studies have also noted neck and tracheal edema after shoulder arthroscopy performed under general anesthesia, with prolonged surgeries and higher fluid volumes [12, 13, 14]. For instance, one study found an average neck circumference increase of 1.17 cm after procedures lasting approximately 147 min with nearly 40 L of fluid used [7]. In contrast, no neck edema was seen in our study, which utilized interscalene block anesthesia and shorter operative times. The neck edema reported in previous studies may be linked to prolonged surgeries, higher irrigation fluid use, and general anesthesia, with laryngeal edema being a common complication of endotracheal intubation (prevalence ranging from 9% to 84%) [7, 12, 13, 14, 15].

Distal peripheral neuropathy is a reported complication in approximately 3% of patients following arthroscopic RCR, with contributing factors potentially including limb manipulation, traction, interscalene brachial plexus blocks, fluid extravasation, postoperative swelling, and prolonged immobilization [5]. Thomasson et al. [5] suggested that proximal nerve injuries are more likely caused by traction and anesthesia, whereas compression, particularly from edema at the elbow and wrist, could lead to distal neuropathies. Medvedev et al. [4] also observed a significantly higher incidence of carpal tunnel syndrome within 1 year after arthroscopic RCR and LR surgeries, attributing it to distal fluid spread and compression in the carpal tunnel. In this study, edema was found to accumulate in the elbow and wrist 24 h postsurgery, while the deltoid region showed no significant increase, reinforcing the notion of distal fluid migration. Postoperative interventions, such as cryotherapy, mobilization, or exercise, may help reduce this fluid buildup and prevent related complications like compressive neuropathies.

### 4.4 Limitations and Strengths

This study has several limitations. First, subcutaneous fluid accumulation was measured only 24 h postoperatively, potentially missing longer‐term fluid dynamics and their effects on outcomes. Additionally, the lack of prolonged surgery times and increased irrigation fluid use in our population may limit the applicability of our findings to those cases. While the MoistureMeterD is reliable for measuring local tissue water in lymphedema patients, its reliability postshoulder arthroscopy remains unestablished. Another limitation is that variations in body types may have influenced the placement of measurement landmarks, despite using a tape measure to standardize the process. Furthermore, the absence of circumference measurements limits our ability to assess overall limb swelling with additional data. Although postoperative subcutaneous pectoral edema was noted after arthroscopic LR, the current follow‐up period is insufficient to determine its long‐term clinical significance. Further research with extended follow‐up is needed to assess whether this edema affects final clinical outcomes.

A major strength of this study is the use of an objective, quantitative method for measuring subcutaneous fluid accumulation, namely the dielectric constant method, which provides precise data on tissue water content. Additionally, the prospective nature of the study ensured that fluid accumulation was tracked at multiple time points, providing a comprehensive understanding of postoperative changes. By including both RCR and LR groups, the study allows for meaningful comparisons between different surgical techniques and their impact on fluid distribution, contributing to the broader understanding of postoperative complications. Moreover, all surgeries were performed by a single experienced surgeon, standardizing the procedure and minimizing variability, which enhances the study's internal validity.

## 5 Conclusion

This study provides insights into the acute accumulation of subcutaneous fluid following arthroscopic shoulder surgery, revealing a significant increase in postoperative pectoral edema in patients who underwent LR, while no such edema was observed in those who underwent RCR. Additionally, both surgical groups exhibited increased subcutaneous fluid levels in the cubital fossa and carpal tunnel, though the correlation between this edema and fluid accumulation during shoulder arthroscopy appears to be limited. While no clinical complications were reported acutely, the statistically significant differences in subcutaneous fluid accumulation between the surgical groups underscore the need for cautious postoperative monitoring. These findings suggest that subcutaneous edema, even when asymptomatic, may serve as an early indicator of delayed or subclinical complications, particularly in high‐risk patients. Thus, while the risks associated with fluid extravasation appear limited, vigilant monitoring and tailored postoperative care protocols remain crucial to mitigating potential complications such as compartment syndrome or venous return impairment. This study provides useful information that can help refine clinical practices and improve patient care outcomes in shoulder arthroscopy.

## Author Contributions

Conceptualization: İ.H.A., Mi.H., and U.K. Methodology: İ.H.A. and U.K. Investigation: İ.H.A., Y.Oğ.A., M.A.T. Formal analysis: İ.H.A., M.A.T. Resources: İ.K. and U.K. Writing – original draft: İ.H.A. Writing – Review and Editing: İ.H.A., Y.Oğ.A., Mi.H., M.A.T., İ.K., and U.K. Visualization: İ.H.A. and Y.Oğ.A. Supervision: İş.K. and U.K. Project administration: İ.H.A. and U.K. All authors had full access to the data in the study and take responsibility for the integrity of the data and the accuracy of the data analysis. All authors meet the authorship criteria as defined by the latest guidelines of the International Committee of Medical Journal Editors (ICMJE). Each author has made substantial contributions to the conception or design of the work, or the acquisition, analysis, or interpretation of data; has drafted the work or revised it critically for important intellectual content; has approved the final version to be published; and agrees to be accountable for all aspects of the work, ensuring that questions related to the accuracy or integrity of any part of the work are appropriately investigated and resolved. All authors are in full agreement with the content of the article and consent to its submission to Orthopedic Surgery.

## Ethics Statement

The study received approval from the Gazi University Ethics Committee on September 2023 and informed consent was obtained from all participants. The procedures used in this study adhere to the tenets of the Declaration of Helsinki.

## Conflicts of Interest

The authors declare no conflicts of interest.
